# Resveratrol Mitigates Sevoflurane-Induced Neurotoxicity by the SIRT1-Dependent Regulation of BDNF Expression in Developing Mice

**DOI:** 10.1155/2020/9018624

**Published:** 2020-02-18

**Authors:** Xiaole Tang, Yilin Zhao, Zhiqiang Zhou, Jing Yan, Biyun Zhou, Xiaohui Chi, Ailin Luo, Shiyong Li

**Affiliations:** Department of Anesthesiology, Tongji Hospital, Tongji Medical College, Huazhong University of Science and Technology, 1095 Jiefang Avenue, Wuhan, 430030 Hubei, China

## Abstract

Various lines of evidence suggest that neonatal exposure to general anesthetics, especially repeatedly, results in neuropathological brain changes and long-term cognitive impairment. Although progress has been made in experimental models, the exact mechanism of GA-induced neurotoxicity in the developing brain remains to be clarified. Sirtuin 1 (SIRT1) plays an important role in synaptic plasticity and cognitive performance, and its abnormal reduction is associated with cognitive dysfunction in neurodegenerative diseases. However, the role of SIRT1 in GA-induced neurotoxicity is unclear to date. In this study, we found that the protein level of SIRT1 was inhibited in the hippocampi of developing mice exposed to sevoflurane. Furthermore, the SIRT1 inhibition in hippocampi was associated with brain-derived neurotrophic factor (BDNF) downregulation modulated by methyl-cytosine-phosphate-guanine–binding protein 2 (MeCP2) and cAMP response element-binding protein (CREB). Pretreatment of neonatal mice with resveratrol nearly reversed the reduction in hippocampal SIRT1 expression, which increased the expression of BDNF in developing mice exposed to sevoflurane. Moreover, changes in the levels of CREB and MeCP2, which were considered to interact with BDNF promoter IV, were also rescued by resveratrol. Furthermore, resveratrol improved the cognitive performance in the Morris water maze test of the adult mice with exposure to sevoflurane in the neonatal stage, without changing motor function in the open field test. Taken together, our findings suggested that SIRT1 deficiency regulated BDNF signaling via regulation of the epigenetic activity of MeCP2 and CREB, and resveratrol might be a promising agent for mitigating sevoflurane-induced neurotoxicity in developing mice.

## 1. Introduction

Every year, millions of children undergo diagnostic or surgical procedures under general anesthesia globally, and the long-term potential neurotoxic effects of general anesthetics (GAs) on the infant brain is a worldwide concern. Solid evidence from rodent and nonhuman primate models has proved that exposure to GAs, especially to those targeting the *γ*-aminobutyric acid type A receptor and/or the N-methyl-D-aspartate receptor, during the brain growth spurt period can cause neuroapoptosis, synaptic dysfunction, and long-term behavioral abnormality [1–10]. Although growing preclinical data has been accumulated, ambiguity still exists in clinical studies [11–15]. A few clinical trials showed that a single brief exposure to GAs during the early life did not increase the risk of neurocognitive impairment [16–21]. However, the secondary analysis of the Mayo Anesthesia Safety in Kids study suggested that exposure to multiple procedures under general anesthesia before the age of 3 was associated with a specific pattern of deficits in neuropsychological tests, which was corroborated in experimental models [22]. Although these findings could not show a direct causality between neuropsychological deficits and GA exposure, they presented some useful hints to further evaluate the effects of GA on the neurodevelopment of children.

BDNF is a pivotal regulator of neuronal survival and synaptic plasticity in the hippocampus and other regions of the developing brain [23, 24]. It has been shown that BDNF plays an important role in the formation of certain types of learning, memory, and mood regulation [25, 26]. Previous studies have shown that neonatal exposure to isoflurane and propofol reduces the production of mature BDNF and thus triggers neuroapoptosis [1, 27–29]. Moreover, a persistent reduction in hippocampal BDNF levels in adult rats that were neonatally exposed to isoflurane results in the insufficient synthesis of synaptic proteins and contributes to the cognitive impairment. This effect is mediated by the occupation of the promoter region of exon IV of BDNF with transcriptional factors such as DNA methyltransferase 1, MeCP2, and histone deacetylase 2 [30]. However, it remains unclear how neonatal isoflurane exposure regulates the activity of these transcriptional factors.

SIRT1 is a nicotinamide-adenine dinucleotide-dependent deacetylase belonging to the class III histone deacetylase family. SIRT1 is indispensable for synaptic plasticity and cognitive function under physiological conditions [31, 32]. Furthermore, activation of SIRT1 with resveratrol, a natural polyphenol agonist of SIRT1, mitigated neuronal injury by regulating the expression of BDNF in animal models of neurodegenerative diseases [32–36]. In addition, resveratrol was also found to mitigate age-related cognitive deficits [37–39]. However, whether SIRT1 is involved in sevoflurane exposure-induced cognition impairment and whether resveratrol can provide neuroprotection against the neurotoxicity of sevoflurane still need to be further investigated.

In the present study, we aimed to examine the role of SIRT1 in long-term cognitive changes induced by neonatal exposure to sevoflurane. Moreover, we tested the hypothesis that resveratrol restored anesthesia-induced SIRT1 reduction and improved long-term cognitive performance by modulating its substrates (including MeCP2 and CREB) and further influencing BDNF signaling.

## 2. Materials and Methods

### 2.1. Mouse Anesthesia and Treatment

C57BL/6J mice at 16-18 days of gestation were purchased from the Laboratory Animal Center of Tongji Medical College (Wuhan, China). The mice were kept in cages in suitable environmental conditions (22–24°C, 12-hour light/dark cycle) and were put on a diet of commercial pellet, without water restrictions. Both female and male pups were used in the studies. All experiments were conducted in accordance with the National Institute of Health *Guide for the Care and Use of Laboratory Animals*. The committee of Tongji Medical College has approved the animal protocol.

**Figure 1:**
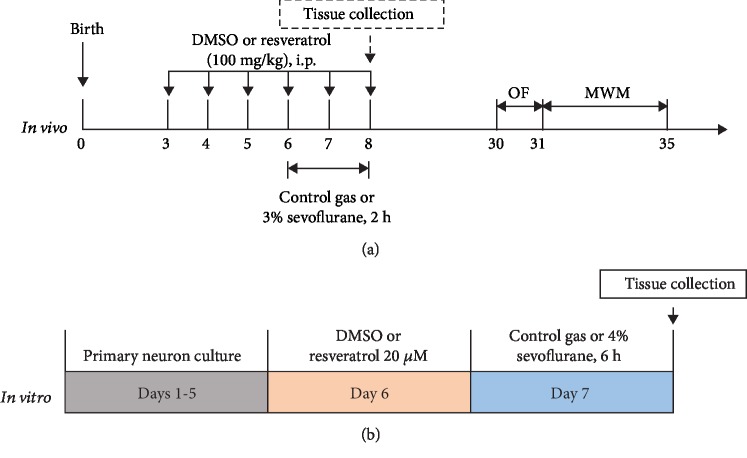
Experiment protocol in vivo and in vitro. (a) *In vivo*: 3-day-old mice were pretreated with DMSO or resveratrol via intraperitoneal injection daily for 6 consecutive days and received control gas exposure or 3% sevoflurane for 2 h from P6 to P8. After exposure, some of the mice were decapitated and tissue samples were collected for RT-PCR and western blotting; the other mice were returned to dams and received the OF test and MWM test from P30 to P35. (b) *In vitro*: after 5-day culture, primary neurons were incubated with DMSO or 20 *μ*M resveratrol followed by 6-hour exposure to control gas or 4% sevoflurane.

As shown in Figure 1(a), the neonatal mice were assigned randomly to four groups: control plus DMSO, control plus resveratrol treatment, sevoflurane plus DMSO, and sevoflurane plus resveratrol. The mice in anesthesia groups received 3% sevoflurane plus 60% oxygen (balanced with nitrogen) 2 h daily form postnatal day (P)6 to P8 in the induction chamber as previous studies described [40–42]. The induction flow rate was 2 L/min for the first three minutes (for induction) and then 1 L/min with the rest of the anesthesia (for maintenance). Mice in the control conditions were separated from dams for the same period of time and exposed to the control gas (60% oxygen balanced with nitrogen). The concentrations of sevoflurane and oxygen were continuously monitored with an infrared probe (Ohmeda S/5 Compact, Datex-Ohmeda, Louisville, CO) during anesthesia. The anesthesia chamber was kept in a homoeothermic incubator to maintain the experimental temperature at 32°C ± 0.5°C. Previous studies have confirmed that the anesthesia with 3% sevoflurane for 2 h did not significantly change pH values, oxygen partial pressure, or carbon dioxide partial pressure in young mice as compared to those in the control group. Therefore, blood gas analysis of the mice was not conducted in our studies. In the intervention studies, 100 mg/kg resveratrol (dissolved in DMSO and diluted with saline) was administrated intraperitoneally (i.p.) on P3, P4, P5, and 1 h prior to isoflurane or control gas exposure from P6 to P8. Mice in the control group received 50 *μ*L DMSO solution (30 *μ*L of DMSO dissolved in 1 mL of saline), which is the vehicle of resveratrol. The number of animals used in each group is reported in Supplementary [Supplementary-material supplementary-material-1].

### 2.2. Harvest of Brain Tissues

Mice were allowed to completely recover from anesthesia. One hour after the end of sevoflurane anesthesia on P8, the mice were euthanized and decapitated. For western blot analysis, the harvested hippocampus tissues were homogenized on ice using RIPA lysis buffer (150 mM sodium chloride, Triton X-100, 0.5% sodium deoxycholate, 0.10% sodium dodecyl sulfate, and 50 mM Tris, pH 8.0) plus 1 mM PMSF, protease inhibitors (Promoter, Wuhan, China), and phosphatase inhibitors (Boster, Wuhan, China). The lysates were collected and centrifuged at 12,000 rpm for 10 min, and total protein was quantified with a BCA assay kit (Boster).

### 2.3. Primary Neurons and Cell Treatment

C57BL/6J mice (The Laboratory Animal Center of Tongji Medical College, China) at 16-18 days of gestation were euthanized with carbon dioxide. Embryonic hippocampi were dissected as previously described [43]. The neurons were digested with 0.05% trypsin for 30 min at 37°C. Then, the dissociated cells were plated at a density of 100,000 cells/well in poly-D-lysine-coated 6-well plates with DMEM/F12 medium (Gibco, Waltham, USA) supplemented with 10% fetal bovine serum (Gibco, USA). Four hours after plating, the medium was replaced with serum-free B27/neurobasal medium. The medium was replaced every 3 days.

The neurons were randomly assigned to four groups: control plus DMSO, control plus resveratrol, sevoflurane plus DMSO, and sevoflurane plus resveratrol, to receive incubation of 0.1% DMSO (the vehicle of resveratrol), 20 *μ*M resveratrol, 0.1% DMSO, and 20 *μ*M resveratrol, respectively, at the sixth day of culture for 24 h. After seven days of plating, the neurons received 4.1% sevoflurane or control gas (21% O_2_, 5% CO_2_, and 70% nitrous oxide) exposure for 6 h (Figure 1(b)).

### 2.4. Neuron Lysis and Protein Quantification

The cells were harvested at the end of the experiments, and neuronal pellets were detergent-extracted on ice using RIPA lysis buffer (150 mM sodium chloride, Triton X-100, 0.5% sodium deoxycholate, 0.10% sodium dodecyl sulfate, and 50 mM Tris, pH 8.0) plus 1 mM PMSF, protease inhibitors (Promoter, Wuhan, China), and phosphatase inhibitors (Boster, Wuhan, China). The lysates were collected and centrifuged at 12,000 rpm for 10 min, and total protein was quantified with a BCA assay kit (Boster).

### 2.5. Reverse Transcriptase Polymerase Chain Reaction (RT-PCR)

Total RNA was extracted from hippocampal tissues with a TRIzol reagent (Invitrogen Life Technologies, Carlsbad, CA, USA) according to the manufacturer's instructions. Isolated RNA (5 *μ*g) was reverse transcribed into cDNA with an RT-PCR kit (TOYOBO, Osaka, Japan). Real-time RT-PCR was carried out as follows: initial denaturation for 1 min at 95°C followed by 40 cycles of 15 s at 95°C, 20 s at 58°C, and 45 s at 72°C. The PCR primers were as follows: Sirt1 forward (5′-ATCGTTACATATTCCACGGTGCT-3′), Sirt1 reverse (5′-CACTTTCATCTTCCAAGGGTTCT-3′); BDNF forward (5′-GCCTCCTCTACTCTTTCTG-3′), BDNF reverse (5′-GGATTACACTTGGTCTCGT-3′). The intensities of the bands were analysed with Image-Pro Plus 6.0 software.

### 2.6. Western Blot Analysis

The harvested brain tissues and cells were subjected to western blotting as described by Narasimhan et al. [44]. In brief, hippocampal tissue lysates (40 *μ*g protein) and primary neuron lysates (20 *μ*g protein) were separated by SDS-PAGE on 10% polyacrylamide gels and transferred to polyvinylidene difluoride membranes. The membranes were incubated with specific primary antibodies, and the antibodies used were SIRT1 (1 : 500, Affinity, OH, USA), BDNF (1 : 500, Affinity, OH, USA), acetyl-MeCP2 (AcMeCP2) (1 : 1000, Millipore, Bedford, MA, USA), MeCP2 (1 : 1000, Cell Signaling Technology, Beverly, MA), phosphorylated CREB (p-CREB) (1 : 1000, ABclonal, Wuhan, China), CREB (1 : 500, Cell Signaling Technology, Beverly, MA), synapsin1 (1 : 5000, Abcam, Cambridge, UK), VGluT1 (1 : 500, Abcam, Cambridge, UK), postsynaptic density 95 (PSD95) (1 : 500, Abcam, Cambridge, UK), and Homer1a (1 : 2000, Abcam, Cambridge, UK). To verify the uniformity of the protein loading and transfer efficiency across the test samples, the membranes were reprobed with GAPDH. Immunoreactive bands were developed with Immobilon Western Chemiluminescent HRP Substrate (Boster), visualized by using an enhanced chemiluminescence system (ChemiDoc, Bio-Rad, USA), and normalized to GAPDH expression.

### 2.7. Coimmunoprecipitation

The hippocampi were dissected from neonatal rats and then homogenized in immunoprecipitation buffer with protease inhibitors and phosphatase inhibitors, and the protein concentration was adjusted to 1 mg/mL. Samples (500 *μ*L) were precleared with nonspecific rabbit IgG (Sigma) followed by adsorption on protein G-Sepharose (Millipore). Immunoprecipitation was performed overnight at 4°C with 5 *μ*g of rabbit anti-SIRT1 (Cell Signaling Technology), anti-AcMeCP2 (Millipore), and anti-CREB1 (Cell Signaling Technology) antibodies, followed by adsorption to protein G-Sepharose. In control experiments, the antibodies were replaced with nonspecific rabbit IgG (Sigma). For western blotting, immunoprecipitates were loaded on 10% SDS-polyacrylamide gels and then transferred onto PVDF membranes. The blots were incubated with specific primary antibodies and then with peroxidase-conjugated species-matched secondary antibodies. The experiments were repeated in triplicate.

### 2.8. Open Field Test

The locomotor and exploratory activities of the animals were monitored by the open field test. The apparatus was a white box with a 50 × 50 cm field surrounded by 40 cm high walls. The mice were placed in the middle of the box and were given 5 min to move freely about the apparatus. Activity in the field was measured using an automated video-tracking system (AVTAS v3.3; AniLab Software and Instruments Co., Ltd., Ningbo, China). The total distance traveled and the average speed were monitored to calculate locomotor activity. After each session, the floor and walls of the field were cleaned with 75% ethanol to eliminate olfactory cues. To avoid experimenter bias, all behavioral experiments were performed by two experimenters who were blind to the experimental protocol. In addition, the data was analysed by another experimenter who was blind to the experimental protocol.

### 2.9. Morris Water Maze Test

The mice (*n* = 8 per group) used for the MWM test were weaned at P22 and tested from P31 to P35. The Morris water maze (MWM) test was performed as previously described [45–47] with small modifications. As shown in Supplementary [Supplementary-material supplementary-material-1], the MWM test was conducted in two separate rooms: test room and computer room. In the test room, there was a round and steel pool (diameter, 120 cm; height, 60 cm), which was filled up with water. Four graphic signals were hung on the walls as visual cues for the navigation of mice in the MWM. Swimming activity of each mouse was tracked via a camera mounted overhead and was analysed by AVTAS v3.3, an automated video-tracking system. Four platforms (platforms ①, ②, ③, and ④) and four quadrants (quadrants 5, 6, 7, and 8) were generated automatically by the system, and platform ② and quadrant 6 were defined as the target platform and target quadrant (Supplementary Figures [Supplementary-material supplementary-material-1] and [Supplementary-material supplementary-material-1]). The data was recorded automatically in a computer, which contains this system and was put in the computer room. By adding powdered milk, the water was rendered opaque. A platform (diametric distance, 10 cm) was placed in the target quadrant (platform ② in quadrant 6) and was 1.0 cm lower than the water level (Supplementary Figures [Supplementary-material supplementary-material-1] and [Supplementary-material supplementary-material-1]). All mice received 5-day training followed by a probe trial. In the training days, the mice were trained to swim to a hidden platform in four trials per day for 5 days (P31-P35). They are allowed to find the platform within 60 s and then have a 15-second stay in the platform. If a mouse failed to find the platform within 60 s, it was guided to the platform and allowed to stay there for 15 s. The mice were tested individually and placed into various quadrants of the pool. The daily order of entry into individual quadrants is randomized, and the mice were allowed to rest at least 30 min before starting the training in the other quadrants. Two hours after the end of the training trials on the fifth day, the probe trial was performed in which the platform is removed from the pool to measure spatial bias. The time spent in searching and mounting the hidden platform in the training trial (escape latency), the distance traversed to reach the hidden platform in the training trial (escape path length), the duration of time spent in each quadrant, and the number of platform crossings in the probe trial were used to assess spatial learning and memory of different group mice. To avoid experimenter bias, all behavioral experiments were performed by two experimenters who were blind to the experimental protocol. In addition, the data was analysed by another experimenter who was blind to the experimental protocol.

### 2.10. Statistics

The data obtained from the biochemistry studies and behavioral tests were presented as the mean ± SEM and were analysed with GraphPad Prism 7.0 (GraphPad Software, CA, USA). Student's *t*-test was used to compare the differences between the control and sevoflurane anesthesia groups. Two-way repeated-measured analysis of variance (ANOVA) was used to evaluate the differences in escape latency and escape path length in the MWM test. Two-way ANOVA followed by the Bonferroni post hoc test was used to evaluate the interaction of groups (control vs. anesthesia) and treatments (resveratrol vs. vehicle) on the amounts of SIRT1, BDNF, p-CREB, CREB, AcMeCP2, MeCP2, synapsin1, VGluT1, PSD95, and Homer1a. The data of escape latency and escape path length obtained from the acquisition training (P31-P35) of the MWM were analysed using a two-way ANOVA (treatment × trial time) with repeated measures (trial days) followed by a Bonferroni post hoc test. The data of time spent in the target quadrant and platform crossing times obtained from probe trial (P35) were analysed using two-way ANOVA (group × treatment) without repeated measures followed by a Bonferroni post hoc test to determine significant differences between experimental groups. *P* value < 0.05 was considered statistically significant.

## 3. Results

### 3.1. Sevoflurane Exposure Inhibited SIRT1 Expression and Decreased BDNF Expression *Ex Vivo* and *In Vitro*

**Figure 2:**
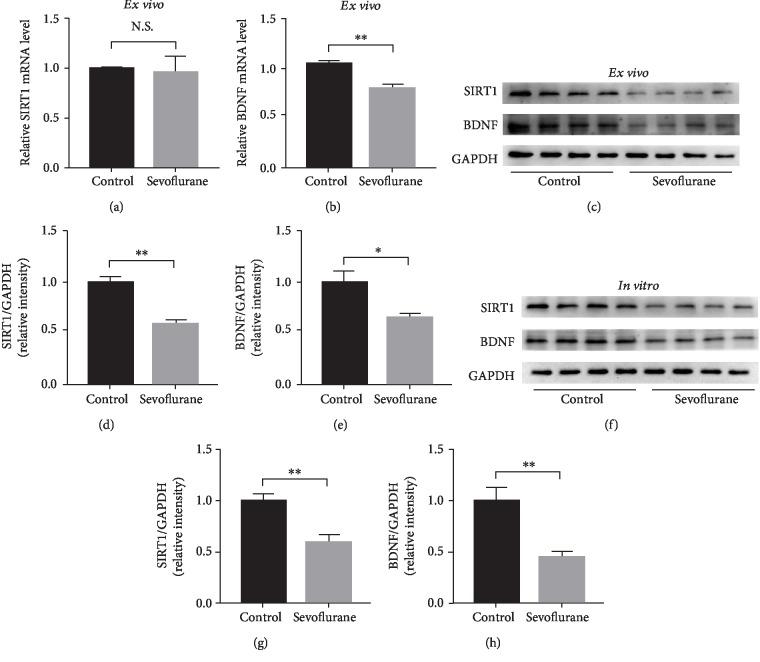
Effect of sevoflurane exposure on the expression of SIRT1 and BDNF *in vivo* and *in vitro*. (a, b) RT-PCR analysis of SIRT1 and BDNF mRNA expression between the control group and the sevoflurane group in hippocampal tissue, respectively (*n* = 3 per group). (c) Representative western blot bands of SIRT1 and BDNF in the neonatal mouse hippocampus (*n* = 4 per group). (d, e) Densitometry quantification of SIRT1 and BDNF expression *ex vivo*, respectively. (f) Representative western blot bands of SIRT1 and BDNF in primary hippocampal neurons (*n* = 4 per group). (g, h) Densitometry quantification of SIRT1 and BDNF expression *in vitro*, respectively. Data are shown as means ± SEM (*n* = 4 per group). ^∗^*P* < 0.05, ^∗∗^*P* < 0.01.

In the *ex vivo* experiment, 6-day-old mice received anesthesia with 3% sevoflurane 2 h daily for 3 days. The mice were sacrificed 1 h after anesthesia, and their hippocampi were harvested. The effects of repeated sevoflurane exposure on SIRT1 and BDNF levels were determined at the mRNA and protein levels. As shown in Figures 2(a) and 2(b), although there was no difference in the expression level of SIRT1 mRNA, BDNF mRNA expression decreased after repeated sevoflurane exposure (*P* < 0.01). At the protein level, both SIRT1 and BDNF expressions were suppressed (*P* < 0.01 and *P* < 0.05, respectively) (Figures 2(c)–2(e)).

In the *in vitro* experiment, the neurons were treated with 4.1% sevoflurane for 6 h on the seventh day of culture. The neurons were lysed to detect SIRT1 and BDNF protein levels by western blotting. As shown in Figures 2(f)–2(h), the sevoflurane anesthesia reduced the protein level of SIRT1 and BDNF protein as compared to the control conditions (*P* < 0.01).

### 3.2. MeCP2 and CREB Expression Changed after Sevoflurane Exposure *Ex Vivo* and *In Vitro*

**Figure 3:**
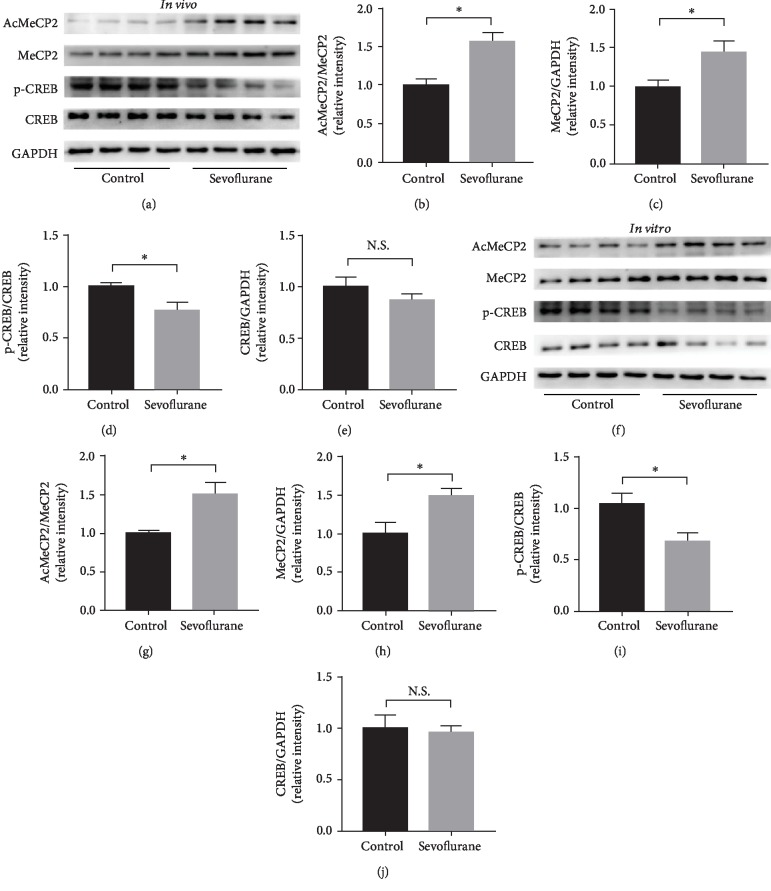
Alterations in MeCP2 and CREB expression. (a) Representative western blot bands of AcMeCP2, total MeCP2, p-CREB, and total CREB in the developing mouse hippocampus. (b–e) Densitometry quantification of AcMeCP2, total MeCP2, p-CREB, and total CREB expression *ex vivo*, respectively. (f) Representative western blot bands of AcMeCP2, total MeCP2, p-CREB, and total CREB in primary hippocampal neurons. (g–j) Densitometry quantification of AcMeCP2, total MeCP2, p-CREB, and total CREB expression *in vitro*, respectively. Data are shown as means ± SEM (*n* = 4 per group). ^∗^*P* < 0.05.

In hippocampus of young mice, the expression of AcMeCP2 as well as that of total MeCP2 was increased (*P* < 0.05) (Figures 3(a)–3(c)) after sevoflurane exposure. There was no significant difference in terms of total CREB expression in hippocampal tissues between the sevoflurane anesthesia and control conditions, while the level of phosphorylated CREB (p-CREB) was declined after repeated sevoflurane exposure (*P* < 0.05) (Figures 3(a), 3(d), and 3(e)).

The quantification of the western blotting results also showed that there was a significant interaction among MeCP2, CREB expression, and sevoflurane exposure in primary neurons. By comparison with the control condition, the sevoflurane anesthesia group displayed significantly elevated levels of AcMeCP2 and total MeCP2 (*P* < 0.05) (Figures 3(f)–3(h)). In line with *in vivo* experiment, sevoflurane exposure also significantly reduced levels of phosphorylated CREB (*P* < 0.05) without the change of total CREB expression (Figures 3(f), 3(i), and 3(j)).

### 3.3. Resveratrol Prevented Sevoflurane-Induced Decreases in SIRT1 and BDNF Expression *Ex Vivo* and *In Vitro*

**Figure 4:**
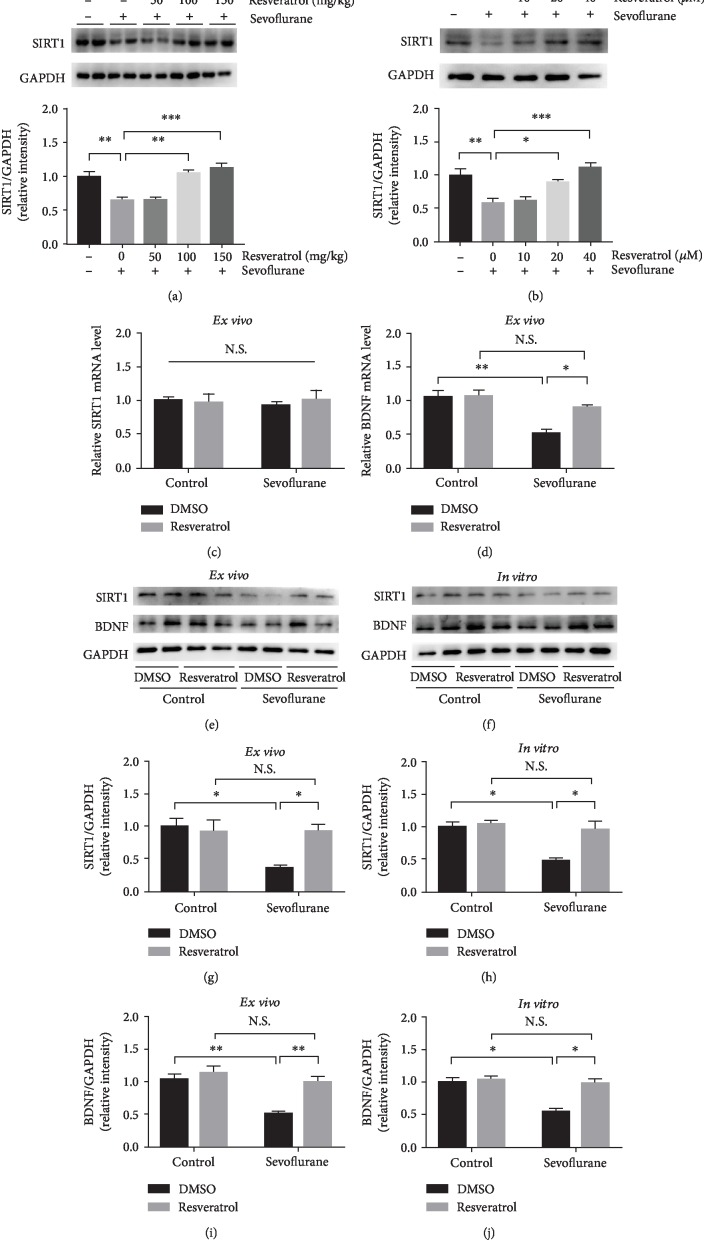
Resveratrol prevented sevoflurane-induced decreases in SIRT1 and BDNF *ex vivo* and *in vitro*. (a) Neonatal mice were pretreated with 50 mg/kg, 100 mg/kg, and 150 mg/kg resveratrol from P3 to P8 and exposed to sevoflurane from P6 to P8. Western blotting was performed to determine the minimum effective dose of resveratrol. (b) Primary hippocampal neurons were pretreated with 10 *μ*M, 20 *μ*M, and 40 *μ*M resveratrol 24 h before exposure to sevoflurane. Western blotting was used to determine the minimum effective concentration of resveratrol. (c, d) RT-PCR results showing SIRT1 and BDNF mRNA expression in the developing mouse hippocampus between groups (control group vs. sevoflurane group) and treatments (DMSO or resveratrol), respectively. (e) Representative western blot bands of SIRT1 and BDNF in the developing mouse hippocampus between groups (control group vs. sevoflurane group) and treatments (DMSO or resveratrol). (f) Representative western blot bands of SIRT1 and BDNF in hippocampal neurons between groups (control group vs. sevoflurane group) and treatments (DMSO or resveratrol). (g, i) Densitometry quantification of SIRT1 and BDNF *ex vivo*, respectively. (h, j) Densitometry quantification of SIRT1 and BDNF protein expression *in vitro*, respectively. Data are shown as means ± SEM (*n* = 4 per group). ^∗^*P* < 0.05, ^∗∗^*P* < 0.01, and ^∗∗∗^*P* < 0.001.

Evidence has shown that resveratrol, a potent SIRT1 activator, is a powerful neuroprotective agent and may play a role in the treatment of neurodegenerative diseases [48]. Based on previous studies [37, 38, 49], three concentrations of resveratrol (50 mg/kg, 100 mg/kg, and 150 mg/kg) were used, and the minimum effective concentration was determined for subsequent experiments. Mice were pretreated with resveratrol via intraperitoneal injection daily from P3 to P6 and 1 h before sevoflurane exposure on P6, P7, and P8. As shown in Figure 4(a), SIRT1 expression was upregulated dose-dependently in resveratrol-treated mice in comparison with that in mice that underwent sevoflurane exposure without resveratrol pretreatment. Although the injection of 50 mg/kg resveratrol did not significantly alter SIRT1 expression, 100 mg/kg and 150 mg/kg resveratrol injection increased SIRT1 expression compared with that in the sevoflurane group (*P* < 0.01 and *P* < 0.001). Thus, we chose 100 mg/kg resveratrol as the dose for the intervention experiment.

There was no difference in the expression of SIRT1 at the RNA level between mice pretreated with resveratrol or DMSO before sevoflurane exposure (Figure 4(c)). However, BDNF expression in mice pretreated with resveratrol was elevated when compared to mice pretreated with DMSO before sevoflurane exposure (*F*_sevoflurane (1, 12)_ = 22.41, *P* = 0.0015; *F*_resveratrol (1, 12)_ = 7.015, *P* = 0.0293; and *F*_sevoflurane∗resveratrol (1, 12)_ = 6.531, *P* = 0.0339) (Figure 4(d)). At the protein level, sevoflurane decreased SIRT1 and BDNF expression in the mice pretreated with DMSO as compared to control conditions. However, in the mice pretreated with resveratrol, sevoflurane did not decrease the expression of SIRT1 (*F*_sevoflurane (1, 12)_ = 7.066, *P* = 0.0209; *F*_resveratrol (1, 12)_ = 4.419, *P* = 0.0573; and *F*_sevoflurane∗resveratrol (1, 12)_ = 7.494, *P* = 0.018) and BDNF (*F*_sevoflurane (1, 12)_ = 16.41, *P* = 0.0016; *F*_resveratrol (1, 12)_ = 13.59, *P* = 0.0031; *F*_sevoflurane∗resveratrol (1, 12)_ = 5.545, *P* = 0.0364) as compared to control conditions (Figures 4(e), 4(g), and 4(i)).

Based on previous studies [50–52], hippocampal neurons were treated with resveratrol for 24 h, and the optimal concentration of resveratrol was selected from10 *μ*M, 20 *μ*M, and 40 *μ*M. As shown in Figure 3(b), exposure to low concentrations of resveratrol (10 *μ*M) did not prevent sevoflurane-induced SIRT1 suppression. In comparison, exposure to high concentrations of resveratrol (20 *μ*M and 40 *μ*M) for 24 h effectively increased SIRT1 expression (*P* < 0.05 and *P* < 0.001). Therefore, intervention group neurons were treated with 20 *μ*M resveratrol for 24 h before sevoflurane anesthesia. In keeping with *ex vivo* experiment, sevoflurane reduced SIRT1 and BDNF levels as compared to the control conditions, and resveratrol blocked the sevoflurane-induced changes in SIRT1 and BDNF levels (*F*_sevoflurane (1, 12)_ = 12.76, *P* = 0.0038; *F*_resveratrol (1, 12)_ = 9.084, *P* = 0.0108; *F*_sevoflurane∗resveratrol (1, 12)_ = 6.665, *P* = 0.024; *F*_sevoflurane (1, 12)_ = 8.63, *P* = 0.0124; *F*_resveratrol (1, 12)_ = 7.61, *P* = 0.0173; and *F*_sevoflurane∗resveratrol (1, 12)_ = 5.17, *P* = 0.0422, respectively) (Figures 4(f), 4(h), and 4(j)).

### 3.4. Resveratrol Reversed the Expression Change of MeCP2 and CREB Induced by Sevoflurane Exposure *Ex Vivo* and *In Vitro*

**Figure 5:**
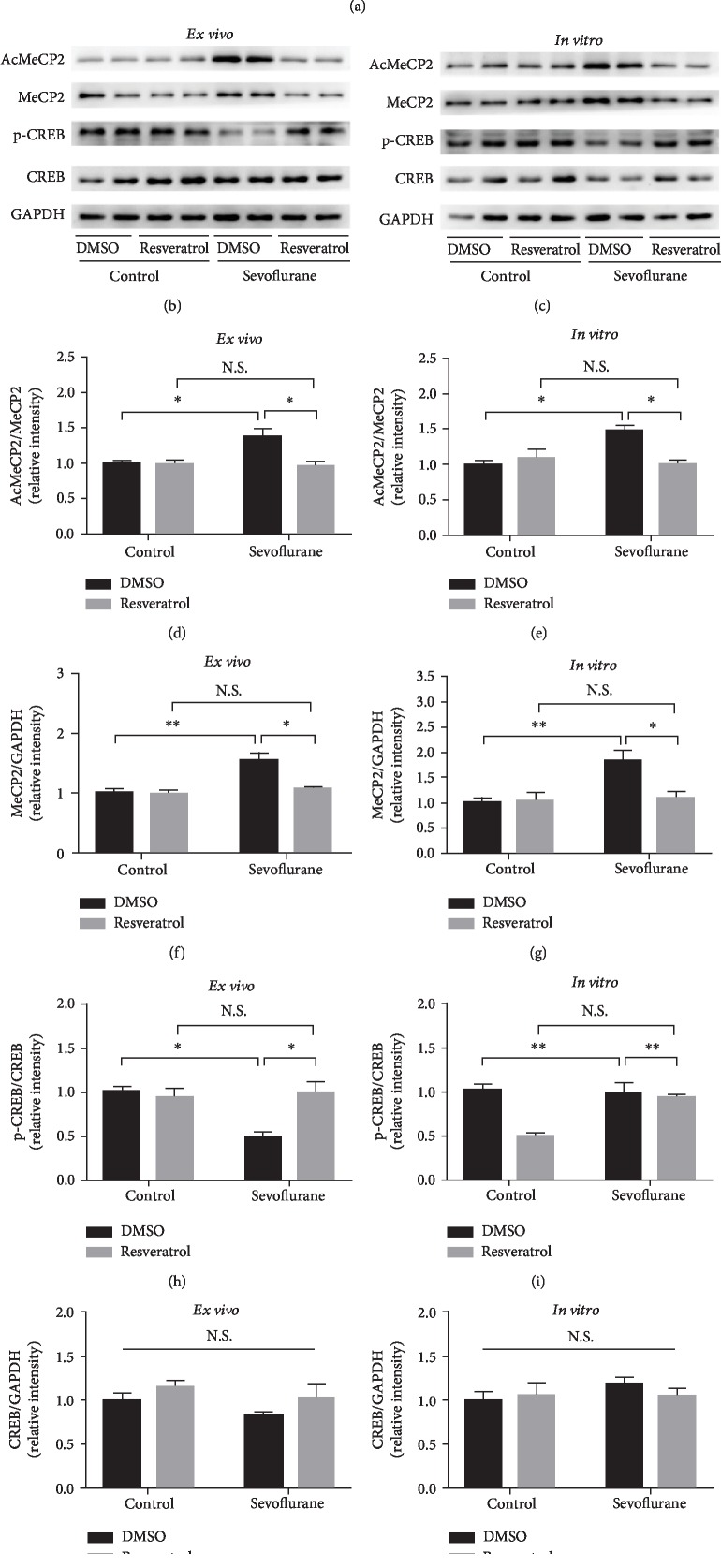
Resveratrol reversed the change in the expression of MeCP2 and CREB induced by sevoflurane exposure *ex vivo* and *in vitro*. (a) Co-IP results for SIRT1, AcMeCP2, and CREB. (b) Representative western blot bands of AcMeCP2, total MeCP2, p-CREB, and total CREB in the developing mouse hippocampus between groups (control group vs. sevoflurane group) and treatments (DMSO or resveratrol). (c) Representative western blot bands of AcMeCP2, total MeCP2, p-CREB, and total CREB in primary hippocampal neurons between groups (control group vs. sevoflurane group) and treatments (DMSO or resveratrol). (d, f, h) Densitometry quantification of AcMeCP2, total MeCP2, p-CREB, and total CREB expression *ex vivo*, respectively. (e, g, i) Densitometry quantification of AcMeCP2, total MeCP2, p-CREB, and total CREB expression *in vitro*, respectively. Data are shown as means ± SEM (*n* = 4 per group). ^∗^*P* < 0.05, ^∗∗^*P* < 0.01.

Previous studies have indicated that SIRT1 could interact with AcMeCP2 and CREB [33, 53]. This finding was also confirmed by our Co-IP results (Figure 5(a)). In the *ex vivo* experiment, resveratrol reversed sevoflurane-induced changes in the expression of MeCP2 and CREB. Notably, following resveratrol administration, AcMeCP2 (*F*_sevoflurane (1, 12)_ = 4.771, *P* = 0.0495; *F*_resveratrol (1, 12)_ = 7.552, *P* = 0.0177; and *F*_sevoflurane∗resveratrol (1, 12)_ = 6.603, *P* = 0.0246) and total MeCP2 (*F*_sevoflurane (1, 12)_ = 13.08, *P* = 0.0035; *F*_resveratrol (1, 12)_ = 8.213, *P* = 0.0142; and *F*_sevoflurane∗resveratrol (1, 12)_ = 7.521, *P* = 0.0179) levels decreased, whereas the p-CREB (*F*_sevoflurane (1, 12)_ = 5.889, *P* = 0.0319; *F*_resveratrol (1, 12)_ = 5.787, *P* = 0.0332; and *F*_sevoflurane∗resveratrol (1, 12)_ = 9.886, *P* = 0.0085) level increased (Figures 5(b), 5(d), and 5(f)). In contrast, the CREB level was not affected by resveratrol administration (Figures 5(b), 5(h), and 5(j)).

Consistently, neurons pretreated with 20 *μ*M resveratrol before sevoflurane exposure also showed decreased expression of AcMeCP2 (*F*_sevoflurane (1, 12)_ = 5.048, *P* = 0.0442; *F*_resveratrol (1, 12)_ = 4.903, *P* = 0.0469; and *F*_sevoflurane∗resveratrol (1, 12)_ = 10.24, *P* = 0.0076) and total MeCP2 (*F*_sevoflurane (1, 12)_ = 13.08, *P* = 0.0035; *F*_resveratrol (1, 12)_ = 8.213, *P* = 0.0142; and *F*_sevoflurane∗resveratrol (1, 12)_ = 6.686, *P* = 0.0238) but increased expression of p-CREB (*F*_sevoflurane (1, 12)_ = 7.038, *P* = 0.0211; *F*_resveratrol (1, 12)_ = 14.32, *P* = 0.0026; and *F*_sevoflurane∗resveratrol (1, 12)_ = 9.537, *P* = 0.0094) by comparison with that in the DMSO pretreated group (Figures 5(c), 5(e), and 5(g)). The expression of CREB did not change with the use of resveratrol (Figures 5(c), 5(i), and 5(k)).

### 3.5. Resveratrol Restored Synaptic Protein Expression *Ex Vivo* and *In Vitro*

**Figure 6:**
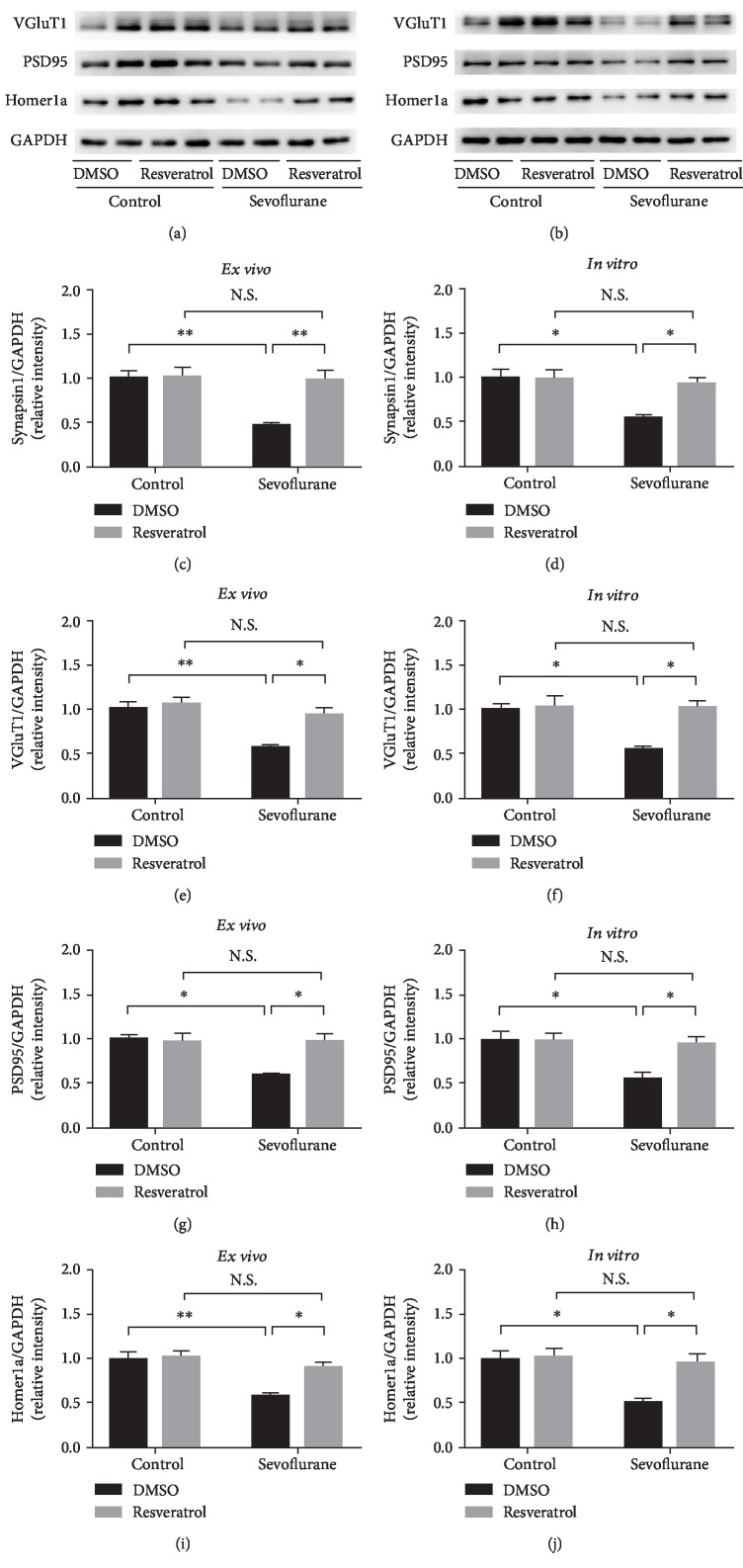
Resveratrol restored synaptic protein expression *ex vivo* and *in vitro*. (a) Representative western blot bands of synapsin1, VGluT1, PSD95, and Homer1a protein in the developing mouse hippocampus between groups (control group vs. sevoflurane group) and treatments (DMSO or resveratrol). (b) Representative western blot bands of synapsin1, VGluT1, PSD95, and Homer1a protein in primary hippocampal neurons between groups (control group vs. sevoflurane group) and treatments (DMSO or resveratrol). (c, e, g, i) Densitometry quantification of synapsin1, VGluT1, PSD95, and Homer1a *in vivo*, respectively. (d, f, h, j) Densitometry quantification of synapsin1, VGluT1, PSD95, and Homer1a *in vitro*, respectively. Data are shown as means ± SEM (*n* = 4 per group). ^∗^*P* < 0.05, ^∗∗^*P* < 0.01.

Synaptic efficacy depends on the presynaptic and postsynaptic compartments. In particular, synapsin1, vesicular glutamate transporters (VGluT1), PSD95, and Homer1a are classical markers of presynaptic and postsynaptic proteins, which play an important role in synaptic activity [54–59]. Previous research has reported that the repeated sevoflurane exposure downregulates synaptic proteins such as postsynaptic protein PSD95 in the hippocampus of developing mice [60, 61]. In the present study, we detect the effect of sevoflurane and resveratrol on the expression of these proteins. Our results indicated that repeated sevoflurane exposure induced a marked decrease in the hippocampal protein levels of synapsin1, VGluT1, PSD95, and Homer1a. However, these downregulations of synaptic proteins were reversed in the presence of resveratrol treatment (synapsin1: *F*_sevoflurane (1, 12)_ = 12.19, *P* = 0.0045; *F*_resveratrol (1, 12)_ = 11.01, *P* = 0.0061; and *F*_sevoflurane∗resveratrol (1, 12)_ = 9.139, *P* = 0.0106; VGluT1: *F*_sevoflurane (1, 12)_ = 15.39, *P* = 0.0020; *F*_resveratrol (1, 12)_ = 9.535, *P* = 0.0094; and *F*_sevoflurane∗resveratrol (1, 12)_ = 5.172, *P* = 0.0421; PSD95: *F*_sevoflurane (1, 12)_ = 6.935, *P* = 0.0218; *F*_resveratrol (1, 12)_ = 5.654, *P* = 0.0349; and *F*_sevoflurane∗resveratrol (1, 12)_ = 6.887, *P* = 0.0222; and Homer1a: *F*_sevoflurane (1, 12)_ = 16.89, *P* = 0.0014; *F*_resveratrol (1, 12)_ = 8.179, *P* = 0.0144; and *F*_sevoflurane∗resveratrol (1, 12)_ = 5.577, *P* = 0.0359) (Figures 6(a), 6(c), 6(e), 6(g), and 6(i)).

Consistently, long-term sevoflurane exposure in primary neurons also led to a significant decrease in the protein levels of presynaptic proteins (synapsin1 and VGluT1), along with the downregulation of postsynaptic proteins (PSD95 and Homer1a) in the PSD fraction. However, these changes in synaptic proteins induced by sevoflurane were significantly attenuated by pretreatment with resveratrol (synapsin1: *F*_sevoflurane (1, 12)_ = 8.505, *P* = 0.0129; *F*_resveratrol (1, 12)_ = 4.853, *P* = 0.0479; and *F*_sevoflurane∗resveratrol (1, 12)_ = 5.719, *P* = 0.034; VGluT1: *F*_sevoflurane (1, 12)_ = 7.698, *P* = 0.0168; *F*_resveratrol (1, 12)_ = 9.36, *P* = 0.0099; and *F*_sevoflurane∗resveratrol (1, 12)_ = 6.711, *P* = 0.0236; PSD95: *F*_sevoflurane (1, 12)_ = 7.643, *P* = 0.0171; *F*_resveratrol (1, 12)_ = 5.108, *P* = 0.0432; and *F*_sevoflurane∗resveratrol (1, 12)_ = 5.5851, *P* = 0.0359; and Homer1a: *F*_sevoflurane (1, 12)_ = 9.21, *P* = 0.0104; *F*_resveratrol (1, 12)_ = 7.464, *P* = 0.0182; and *F*_sevoflurane∗resveratrol (1, 12)_ = 5.892, *P* = 0.0319) (Figures 6(b), 6(d), 6(f), and 6(j)).

### 3.6. Spatial Memory Deficits Induced by Early Repeated Exposure to Sevoflurane Were Alleviated by Treatment with Resveratrol

**Figure 7:**
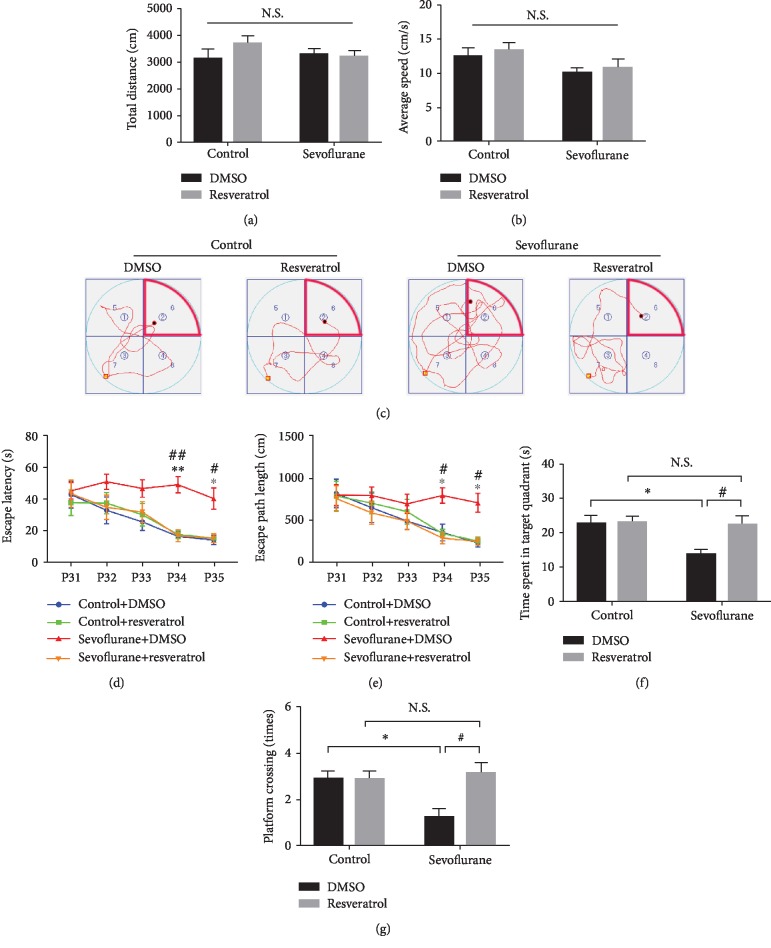
Motor function and spatial memory changes induced by sevoflurane exposure and pretreatment with resveratrol. (a) Total distance traveled by four groups of mice in the open field test. (b) The average speed traveled by four groups of mice in the open field test. (c) Representative swimming paths of the mice in the four experimental groups obtained from the training in quadrant 7 on P34. The mice were placed in the water facing the pool wall at the yellow point in quadrant 7 on the fourth day of training; the swimming path of mice was finished at the black point. Quadrant 6 is the target quadrant (marked by red lines), and platform ② is the target platform. (d) The escape latency in the MWM plotted against the training days. (e) The escape path length in the MWM plotted against the training days. (f) Analysis of the time spent in each quadrant during the probe trial of the MWM. (g) The platform crossing times during the probe trial of the MWM test. Data are shown as means ± SEM (*n* = 8 per group). ^∗^*P* < 0.05, ^∗∗^*P* < 0.01, control+DMSO vs. sevoflurane+DMSO; ^#^*P* < 0.05, ^##^*P* < 0.01, sevoflurane+resveratrol vs. sevoflurane+DMSO.

To determine the long-term cognitive effects of repeated isoflurane exposure and resveratrol treatment, the MWM test was used to detect the spatial memory of the different groups of mice on P31. Before the MWM test, the motor function of young mice was measured by the open field test. As displayed in Figures 7(a) and 7(b), the total distance traveled and average speed did not differ among the four groups of mice. In the MWM test, all groups of mice learned to locate a hidden platform, as evidenced by the decreasing escape latencies (*F*_time (4, 112)_ = 9.702, *P* < 0.001) and escape path length (*F*_time (4, 112)_ = 9.503, *P* < 0.001) over the training period, and the difference was observed between the groups during the same day (*F*_treatment (3, 28)_ = 9.693, *P* < 0.001; *F*_treatment (3, 28)_ = 4.877, *P* < 0.01) (Figure 6(c)). A post hoc test (Bonferroni) showed that, compared with control mice, the mice exposed to sevoflurane required longer escape latency and escape path length to locate the position of the platform in the MWM pool on P34 (*P* < 0.01, *P* < 0.05, respectively) and P35 (*P* < 0.05, *P* < 0.05, respectively) (Figures 7(d) and 7(e)). The mice that were exposed to sevoflurane, compared to control mice, also spent less time in the target quadrant (*F*_sevoflurane (1, 28)_ = 5.455, *P* = 0.0269; *F*_resveratrol (1, 28)_ = 4.97, *P* = 0.034; and *F*_sevoflurane∗resveratrol (1, 28)_ = 4.428, *P* = 0.0445) (Figure 7(f)) and crossed the platform fewer times (*P* < 0.05) (Figure 7(g)).

On the other hand, the Bonferroni post hoc test showed that, compared with sevoflurane mice, the mice pretreated with resveratrol before sevoflurane required shorter escape latency and escape path length to locate the position of the platform in the MWM pool on P34 (*P* < 0.01 and *P* < 0.05, respectively) and P35 (*P* < 0.05 and *P* < 0.05, respectively), longer time in the target quadrant (*F*_sevoflurane (1, 28)_ = 5.455, *P* = 0.0269; *F*_resveratrol (1, 28)_ = 4.97, *P* = 0.034; and *F*_sevoflurane∗resveratrol (1, 28)_ = 4.428, *P* = 0.0445) and crossed the platform more times (*P* < 0.05) (Figures 7(c)–7(g)).

## 4. Discussion

In the present study, we found that SIRT1 deficiency was associated with prolonged cognitive impairment in developing mice following repeated sevoflurane exposure. Sevoflurane-induced SIRT1 inhibition led to the inactivation of MeCP2 and CREB, two critical epigenetic regulators of BDNF, and then reduced the expression of BDNF in the hippocampus. Pretreatment with resveratrol, an agonist of SIRT1, almost restored the expression of SIRT1, increased the epigenetic regulation of MeCP2 and CREB, promoted the expression of BDNF, and consequently mitigated the cognitive deficits. Collectively, our findings showed that SIRT1 deficiency modulated BDNF signaling by influencing the epigenetic activity of MeCP2 and CREB and suggested a promising method for mitigating the neurotoxicity induced by sevoflurane in developing mice.

BDNF has been shown to regulate the survival and the differentiation of neurons and dynamically modulate the synaptic plasticity in the developing brain [23, 24, 34]. BDNF deficiency in the CA1 of the hippocampus led to impaired long-term potentiation and synaptic structure, and administration of recombinant BDNF almost reversed these pathological effects [23, 62]. Several lines of evidence indicated that impaired BDNF signaling was associated with increased neuroapoptosis and disrupted synaptic components in the neonatal rodent following exposure to commonly used GAs [27–29, 62]. In agreement with the aforementioned findings, our data showed that sevoflurane downregulated BDNF in the hippocampi of neonatal mice. BDNF played an important role in synaptic plasticity, partially owing to the regulation of the local synthesis of proteins, including the presynaptic and postsynaptic molecules [24, 63]. As expected, we found that sevoflurane-induced BDNF reduction resulted in decreased levels of the presynaptic components (synapsin1 and VGluT1) and the postsynaptic component (PSD95). Surprisingly, we also found that Homer1a, a key neuroprotective endogenous molecule, was downregulated by sevoflurane. As is known, Homer1a belongs to the postsynaptic density family [64], but it is unknown whether the change of Homer1a is related to the decrease of BDNF in the present study. In addition, the present and previous studies demonstrated that upregulated expression of BDNF mitigated GA-induced neurotoxicity in the developing brain [30, 65]. As is known, BDNF can be produced in neurons, astrocytes, and microglia in the brain. Previous studies showed that neonatal exposure to isoflurane induced BDNF reduction in astrocytes and then promoted neuronal loss [66–68]. In our animal models, we only measured the gross effect of sevoflurane on the expression of BDNF in the developing brain, so we could not find the precise resource of BDNF. Taking into account the experiment of cultured neurons, we cautiously indicated that sevoflurane exposure reduced the expression of BDNF in developing neurons. However, we cannot rule out the possibility that sevoflurane may change the production of BDNF from astrocytes or microglia. These direct and indirect evidences support the notion that the inhibition of neuronal BDNF signaling contributes to the sustained neurobehavioral abnormality in experimental models with repeated exposure to GAs.

The biosynthesis of BDNF can be modulated at different levels. Recently, increasing attention has been paid to the epigenetic reprogramming of BDNF because this regulation mechanism takes part in activity-dependent synaptic function and the crosstalk of neuronal circuits [69, 70]. As aforementioned, GAs inhibited the expression of BDNF when administered at the peak of brain development, and an increasing number of studies had been conducted to explore the mechanism by which GAs regulated the expression of BDNF [1, 30, 71]. In light of the widespread use of sevoflurane in current pediatric anesthesia, we attempted to explore how sevoflurane influenced the expression of BDNF in the developing brain in the present study. We found that the level of acetylated MeCP2 is increased and the level of phosphorylated CREB is decreased in the hippocampi of developing mice following repeated exposure to sevoflurane. As is known, MeCP2 and CREB are two key transcriptional factors that regulate the expression of BDNF in the central nervous system [70]. Their abnormal activity led to BDNF reduction and then impaired the biosynthesis of synaptic proteins [69, 72] in our study. Furthermore, we found that resveratrol, an agonist of SIRT1, almost normalized the sevoflurane-induced changes of MeCP2 and CREB in the hippocampus. Indeed, we found that SIRT1 was inhibited by isoflurane, another inhalational anesthetics, in neonatal rats in our previous study [73]. Therefore, we examined the effect of sevoflurane on the expression of SIRT1 in the hippocampus and did confirm that SIRT1 was reduced by sevoflurane in the current study. It was reported that SIRT1 has been strongly implicated in normal synaptic plasticity and cognitive function [32, 53] by regulating the level of acetylated MeCP2 and the transcriptional activity of CREB in the brain [33, 34]. However, it is unclear whether sevoflurane-induced SIRT1 inhibition modulates the expression of BDNF by inhibiting the activity of MeCP2 and CREB. Together, these findings suggest that SIRT1 reduction and inhibited activity of MeCP2 and CREB are related to sevoflurane-induced decreased BDNF in the developing hippocampus.

SIRT1, a NAD-dependent class III histone deacetylase (HDAC), is involved in various physiological and pathophysiological processes, including neurodevelopment, aging, metabolism, inflammation, and cancer [35]. Its substrates include histone proteins as well as nonhistone proteins [74]. In the present study, we hypothesized that there was a link between the SIRT1 inhibition and abnormal activity of MeCP2 and CREB in developing mice following sevoflurane exposure. To test this hypothesis, we pretreated the neonatal mice with resveratrol before sevoflurane anesthesia. We found that resveratrol pretreatment rescued SIRT1 inhibition, reduced the level of acetylated MeCP2, and increased the levels of phosphorylated CREB and BDNF in the hippocampal tissue and in cultured hippocampal neurons. Furthermore, our coimmunoprecipitation experiment confirmed that SIRT1 could directly bind to MeCP2 or CREB, respectively. CREB-binding protein (CBP) was reduced by sevoflurane in neonatal rats [65]. Similar findings were observed in another study in which general anesthesia (midazolam-nitrous oxide-isoflurane) for 6 hours resulted in CBP reduction in neonatal rats at 24 hours after the treatment ended [71]. In the present study, we found that CBP was not likely correlated with the expression of SIRT1; therefore, the data of CBP was not present. In previous neurodegenerative models, SIRT1 was involved in the regulation of miR134 and transducers of regulated CREB1 (TORC1) and influenced the expression of BDNF [33, 34]. We found that SIRT1 was involved in the regulation of the level of phosphorylated CREB but did not further investigate the potential mechanism by which SIRT1 mediated changes in the transcriptional activity of CREB to affect the expression of BDNF, which is a limitation of our study.

Given the indispensable role of BDNF in GA-induced developmental neurotoxicity, it is essential to explore feasible therapeutic methods for rescuing the expression of BDNF. Several studies found that the increased expression of some class I and class II HADCs was involved in suppressing the expression of BDNF triggered by GA exposure during early life [30, 65, 71, 75, 76]. Theoretically, inhibitors of HDACs might be useful. Strikingly, this hypothesis was supported by a recent study showing that MS-275, a selective inhibitor of class I histone deacetylases (HDAC2, HDAC3, and HDAC8) [77], and sodium butyrate restore GA-induced synaptic alterations along with BDNF alterations [65, 71]. However, it has been reported that inhibitors of class I HDACs have intrinsic toxic side effects and that there is thus a small therapeutic window, which prevents their application in the clinical setting [78]. In the present study, we found that resveratrol pretreatment almost restored the expression of SIRT1 and increased the expression of BDNF by influencing the transcriptional factors MeCP2 and CREB. Resveratrol (3,4′,5-trihydroxystilbene (C_14_H_12_O_3_)) is a natural polyphenolic nutraceutical that has pleiotropic properties, including antioxidation, anti-inflammation, antiapoptosis, and anticancer properties in multiple organs under a wide variety of pathological conditions [79]. It has been reported that supplementation with resveratrol improves memory performance in association with increased hippocampal function connectivity in elderly adults [80]. Besides being known as an agonist of SIRT1, resveratrol also acts as an inhibitor of the mammalian target of rapamycin (mTOR) [81, 82]. Amounts of studies implied that resveratrol could influence autophagy, oxidative stress, and inflammation through the mTOR pathway [49, 82–84]. Additionally, the mTOR pathway was found to take part in the regulation of dendritic growth, synapse formation, and synaptic protein expression [85–87]. It was reported that general anesthetics, including sevoflurane, induced developmental neuronal injury by activating the mTOR pathway [87–90]. In light of the fact that mTOR and BDNF interact in a complicated way in physiological or pathological settings [91, 92], we did not explore the crosstalk between BDNF and mTOR signaling in the sevoflurane-induced injury in the present study. Therefore, it needs to be further investigated whether mTOR signaling is involved in the protection of resveratrol against sevoflurane-induced impairment.

To our knowledge, we have provided the first evidence that resveratrol pretreatment mitigates sevoflurane-induced developmental neurodegeneration and improves long-term cognitive function. Although the efficacy, safety, and pharmacokinetics of resveratrol have been documented in more than 200 clinical trials, there is not yet any trial on its potential to prevent GA-induced neurotoxicity. We hypothesize that resveratrol might be a promising medication for preventing the noxious effect of GA in the developing brain. Although the clinical use of resveratrol is documented in stroke and other neurodegenerative diseases, its poor bioavailability and rapid metabolism may limit its use [93, 94]. Therefore, longer-acting SIRT1 agonists more readily absorbed will be more clinically relevant.

## 5. Conclusions

Taken together, our study suggested that SIRT1 reduction was associated with sevoflurane-induced neurotoxicity. SIRT1 deficiency increases MeCP2 acetylation and decreases CREB phosphorylation, thus resulting in the downregulation of BDNF in the hippocampus of the developing brain. Also, pretreatment with resveratrol mitigates the sevoflurane-induced changes of SIRT1, MeCP2, and CREB in the hippocampus and improves the prolonged neurobehavioral performance in the developing model. Our findings proposed a new mechanism of sevoflurane-induced neurotoxicity in the developing brain and suggested that these long-term cognitive deficits could be mitigated by activating SIRT1.

## Data Availability

The data used to support the findings of this study are included within the article.

## Supplementary Materials

Supplementary Materials Supplementary Table 1: study population. Supplementary Figure 1: the rooms and apparatus used in the MWM test and the division of quadrants and platforms. (A) The rooms and apparatus used in the MWM test. The MWM test was conducted in two separate rooms: test room and computer room. In the test room, there was a round and steel pool, which was filled up with water. Four graphic signals were hung on the walls as visual cues for the navigation of mice in the MWM. Swimming activity of each mouse was tracked via a camera mounted overhead and was analysed by AVTAS v3.3, an automated video-tracking system. The data was recorded automatically in a computer, which contains this system and was put in the computer room. (B) The background of the swimming path recorded by the video-tracking system. By adding powdered milk, the water was rendered opaque. A platform (diametric distance, 10 cm) was placed in the target quadrant and was 1.0 cm lower than the water level. (C) The quadrants and platforms divided automatically by the video-tracking system. Four platforms (platforms ①, ②, ③, and ④) and four quadrants (quadrants 5, 6, 7, and 8) were generated automatically, and platform ② and quadrant 6 were defined as the target platform (marked by a black circle) and target quadrant (marked by red line), respectively. (D) The recorded swimming path shown in the manuscript without background. The target quadrant was marked by a red line.
