# Supplementary material for: Resveratrol Mitigates Sevoflurane-Induced Neurotoxicity by the SIRT1-Dependent Regulation of BDNF Expression in Developing Mice
Source: Oxid Med Cell Longev. 2020 Feb 18;2020:9018624. doi: 10.1155/2020/9018624 (PMC7049870; doi:10.1155/2020/9018624)
Supplement: Supplementary Materials — Supplementary Table 1: study population. Supplementary Figure 1: the rooms and apparatus used in the MWM test and the division of quadrants and platforms. (A) The rooms and apparatus used in the MWM test. The MWM test was conducted in two separate rooms: test room and computer room. In the test room, there was a round and steel pool, which was filled up with water. Four graphic signals were hung on the walls as visual cues for the navigation of mice in the MWM. Swimming activity of each mouse was tracked via a camera mounted overhead and was analysed by AVTAS v3.3, an automated video-tracking system. The data was recorded automatically in a computer, which contains this system and was put in the computer room. (B) The background of the swimming path recorded by the video-tracking system. By adding powdered milk, the water was rendered opaque. A platform (diametric distance, 10 cm) was placed in the target quadrant and was 1.0 cm lower than the water level. (C) The quadrants and platforms divided automatically by the video-tracking system. Four platforms (platforms ①, ②, ③, and ④) and four quadrants (quadrants 5, 6, 7, and 8) were generated automatically, and platform ② and quadrant 6 were defined as the target platform (marked by a black circle) and target quadrant (marked by red line), respectively. (D) The recorded swimming path shown in the manuscript without background. The target quadrant was marked by a red line. [file 9018624.f1.pdf]

Table 1. Study population

Animals	Groups	Sample size	Animals died due to exposure		Survival anmials	Read-out (number of mice)
			During exposure	Recovery period		
Neonatal mice (n =172)	Control + DMSO	n = 39	n = 0	n = 0	n = 39	PCR (3)<br>WB (24)<br>CO-IP (4)<br>Behavioral tests (8)
	Control + resveratrol	n = 27	n = 0	n = 0	n = 27	PCR (3)<br>WB (12)<br>CO-IP (4)<br>Behavioral tests (8)
	Sevoflurane + DMSO	n = 57	n = 5	n = 13	n = 39	PCR (3)<br>WB (24)<br>CO-IP (4)<br>Behavioral tests (8)
	Sevofluane + resveratrol	n = 49	n = 3	n = 7	n = 39	PCR (3)<br>WB (24)<br>CO-IP (4)<br>Behavioral tests (8)

Supplementary 9018624V2.

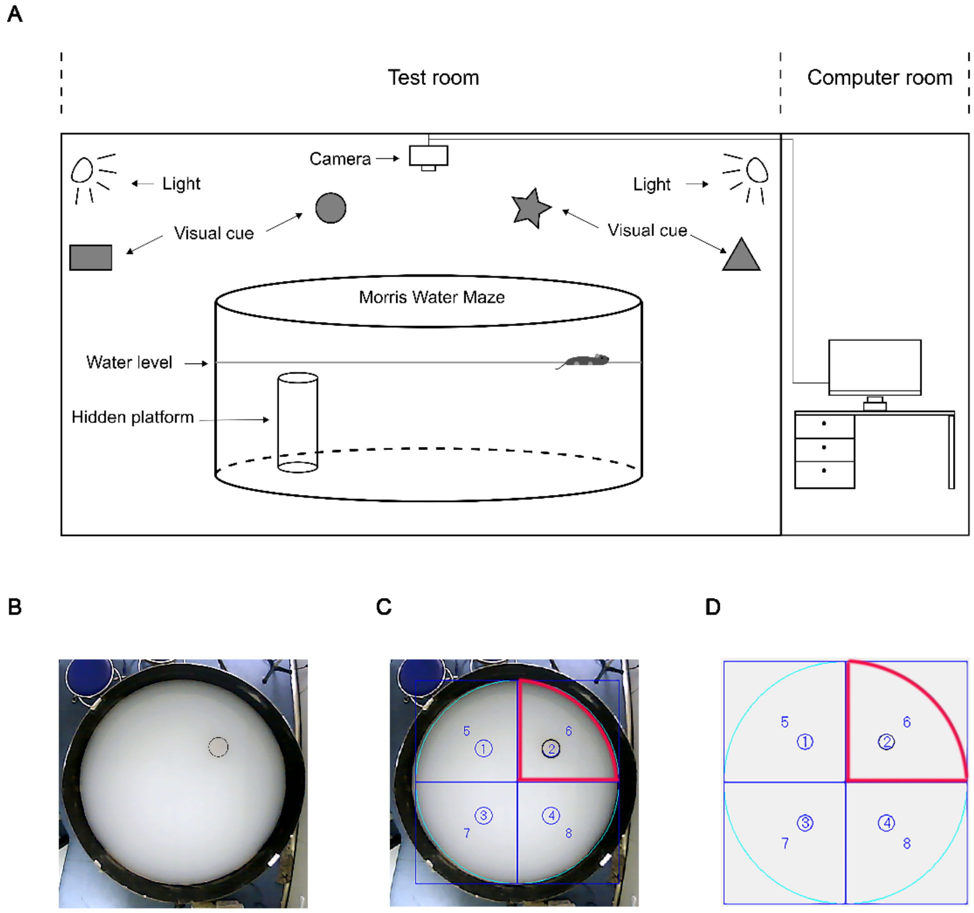

Figure. 1 The rooms and apparatus used in MWM test and the division of quadrants and platforms. (A) The rooms and apparatus used in MWM test. MWM test was conducted in two separate room, test room and computer room. In the test room, there was a round and steel pool, which was filled up with water. Four graphic signals were hung on the walls as visual cues for the navigation of mice in the MWM. Swimming activity of each mouse was tracked via a camera mounted overhead, and was analysed by AVTAS v3.3, an automated video-tracking system. The data was recorded automatically in a computer, which containing this system and put on the computer room. (B) The background of the swimming path recorded by the video-tracking system. By adding powdered milk, the water was rendered opaque. A platform (diametric distance, 10 cm) was placed in the target quadrant and was 1.0 cm lower than the water level. (C)The quadrants and platforms divided automatically by the video-tracking system. Four platforms (platform ①, ②, ③, ④) and four quadrants (quadrant 5, 6, 7, 8) was generated automatically, and platform ② and quadrant 6 was

defined as the target platform (marked by black circle) and target quadrant (marked by red line). (D) The recorded swimming path shown in the manuscript without background. The target quadrant was marked by red line.
